# Reestablishment and Conservation Implications of the Milu Deer Population in Poyang Lake

**DOI:** 10.3390/ani15233446

**Published:** 2025-11-29

**Authors:** Zhibin Cheng, Zhenyu Zhong, Bin Xiong, Xinghua Zhong, Jialiang Ma, Daoli Liu, Chenmiao Feng, Qingyun Guo, Qingxun Zhang, Jiade Bai, Kun Cheng

**Affiliations:** 1 College of Wildlife and Protected Areas, Northeast Forestry University, Harbin 150040, China; czb@milupark.org.cn (Z.C.); 13026556767@163.com (J.M.); 2 Beijing Milu Ecological Research Center, Beijing 100076, China; zzy@milupark.org.cn (Z.Z.); fengmiao1988@163.com (C.F.); guoqingyun1987@126.com (Q.G.); zhangqingxun1990@126.com (Q.Z.); 3 Beijing Biodiversity Conservation Research Center, Beijing 100076, China; 4 Yongxiu County Forestry Bureau, Jiujiang 330304, China; jxyxcyg@163.com; 5 Yugan County Forestry Bureau, Shangrao 335100, China; hhj36@163.com; 6 Poyang County Forestry Bureau, Shangrao 333199, China; 18370367119@139.com

**Keywords:** reintroduction, *Elaphurus davidianus*, deer, endangered species, wetland, in situ conservation

## Abstract

Reintroduction has been recognized as a vital approach to protecting and restoring wild populations of rare and endangered species. Although numerous reintroduction programs have been launched worldwide, in-depth studies on population establishment, dynamics, distribution patterns, and challenges remain limited. The Milu (*Elaphurus davidianus*), endemic to China and once declared Extinct in the Wild by the IUCN, has become one of the most successful examples of global endangered species reintroduction since the initiation of its recovery program in 1985. Focusing on the reintroduction project in the Poyang Lake basin—China’s largest freshwater lake—this study analyzed 14 years of monitoring data to examine the processes of population re-establishment, demographic trends, and spatiotemporal distribution of the species, while also identifying major ongoing challenges. Findings reveal the following: (1) the acclimatization process was essential for 10 individuals first reintroduced in 2013, and then 30 individuals joined to the growing population, followed by the largest-scale release of 47 in 2018, which finally led to eight consecutive years of natural reproduction, with a theoretically estimated population of 89 individuals until August 2025; (2) among 1697 individual sightings in 238 monitoring events, 952 records can be distinguished between male and female individuals (sex ratio = 1:1.63), yielding a mean herd size of 7.28 ± 0.68 individuals per sighting event—although numbers rose slightly in 2024, a declining trend was observed from 2019 to 2023 (from 11.26 to 5.13); (3) the Milu have expanded their range across eastern Poyang Lake, demonstrating a behavioral patterns of migrating out of the lake zone during flood seasons and returning in dry season; (4) the restoration of the Milu population is a systematic endeavor, in which government support and the participation of conservation professionals, scientists, volunteers, media, and the public are critical factors; and (5) ongoing threats such as human disturbance (e.g., habitat degradation) and climate change continue to jeopardize long-term population viability. This study offers important scientific insights into the recovery of endangered wildlife globally and underscores the essential role of governmental commitment, long-term monitoring, and multi-stakeholder collaboration in species reintroduction efforts.

## Simple Summary

The Milu (*Elaphurus davidianus*) is a species endemic to China, listed as Extinct in the Wild on the IUCN Red List of Threatened Species. Since the initiation of reintroduction programs in 1985, wild populations have been successfully reestablished, making it one of the most successful cases of endangered species reintroduction worldwide. This study, focusing on the reintroduction of Milu in Poyang Lake—China’s largest freshwater lake—provides a detailed analysis of the 14-year process of reestablishing a wild population in this basin. It examines the current status of the Milu population, its spatiotemporal distribution, and the challenges faced. The research aims to provide a scientific reference for Milu conservation and also for restoring wild populations of other endangered wildlife species.

## 1. Introduction

Reintroduction of species is one of the most important approaches for the conservation of rare and endangered wildlife and the restoration of their ecosystems [1]. Species reintroduction refers to the process of releasing individuals of a wild species—which has become extinct in its original distribution range but survives in ex situ conservation facilities or other regions—back into its natural habitat [2]. Since the reintroduction of North American wapiti (*Cervus canadensis*) from Yellowstone National Park into other states of USA in 1892, the number of wildlife reintroduction projects implemented worldwide has increased significantly [3]. By 2021, records indicated that more than 1000 reintroduction projects had been carried out covering various taxonomic groups including invertebrates, amphibians, reptiles, birds, and mammals [4,5,6,7,8,9,10,11]. Among these, projects involving species such as the Eurasian lynx *(Lynx lynx*) in Europe [12], the Eurasian beaver (*Castor fiber*) [13], and the gray wolf (*Canis lupus*) in North America [14] have achieved remarkable success, re-establishing self-sustaining wild populations.

As an important component of ecological civilization construction, China has conducted numerous wildlife release programs and made significant progress. Since the 1990s, China has successively carried out reintroduction projects for several endangered species, including the Milu (*Elaphurus davidianus*) [15], giant panda (*Ailuropoda melanoleuca*) [16], red-crowned crane (*Grus japonensis*) [17], Przewalski’s horse (*Equus ferus*) [18], crested ibis (*Nipponia nippon*) [19], Chinese alligator (*Alligator sinensis*) [20], Burmese python (*Python bivittatus*) [21], forest musk deer (*Moschus berezovskii*) [22], and Francois’ langur (*Trachypithecus francoisi*) [23]. These efforts have contributed significantly to rescuing rare and endangered wildlife and restoring ecosystems. However, as China started relatively late in the reintroduction of artificially bred rare and endangered species, the practice is still in an exploratory stage. There is an urgent need to learn from successful reintroduction cases to gain valuable experience.

The Milu, endemic to China, is a national first-class protected wildlife, and classified as Extinct in the Wild on the IUCN Red List of Threatened Species. As a typical wetland species, it was historically widely distributed across wetland basins within 18–45° N and 110–130° E in China, particularly in the Yangtze River and Yellow River basins [24]. Due to habitat loss, hunting, and historical turmoil, the species became extinct in China around 1900. All existing Milu worldwide are descendants of 18 individuals preserved at Woburn Abbey in the United Kingdom in the late 19th century [25]. Between 1985 and 1987, China reintroduced 38 and 39 individuals from the UK to the Beijing Milu Ecological Research Center and the Jiangsu Dafeng Milu National Nature Reserve, respectively, initiating conservation and recovery efforts that have since helped restore the population. In 1993 and 1994, 64 Milu were initially reintroduced into the Shishou Tianezhou Oxbow of the Yangtze River for acclimatization, ultimately leading to the successful establishment of a wild population in Shishou, Hubei. As of December 2020, Milu in China were distributed across 83 sites, with a total population of around 9000 individuals. Among these, four wild populations—located in Jiangsu Dafeng, Jiangsu Yancheng, Hubei Shishou, and Hunan East Dongting Lake—numbered 2765 individuals [26].

Poyang Lake in Jiangxi Province, China’s largest freshwater lake and a wetland of international importance, was historically a core distribution area for the Milu, yet extensive reclamation and human expansion led to the local extinction of large terrestrial mammals (e.g., Milu) centuries ago [25]. Although China has conducted over ten reintroduction initiatives for Milu—all successful except the Mulan Weichang attempt—existing studies have largely emphasized short-term studies in regions such as Jiangsu Dafeng and Hunan Dongting Lake [27,28], leaving significant gaps in understanding key reintroduction processes like site selection, rewilding methods, and long-term population dynamics. In particular, there is a scarcity of systematic research on achieving sustainable population recovery in human-dominated lake ecosystems such as Poyang Lake, where challenges like habitat fragmentation and human–wildlife conflict prevail. Utilizing long-term monitoring data from the 2012–2025 Poyang Lake Milu Reintroduction Project, this study provides the first comprehensive analysis of adaptive rewilding strategies, post-release population dynamics, and spatial distribution in the basin. It addresses central scientific questions including: (1) how to reestablish an endangered species population in a densely human-populated lake environment; (2) the population status and distribution of Milu in Poyang Lake over the 14-year establishment phase; (3) key factors influencing reintroduction success; and (4) ongoing challenges facing the reconstructed population. The findings offer a scientific foundation for restoring wild populations in Poyang Lake and informing future reintroductions of rare and endangered wildlife species.

## 2. Materials and Methods

### 2.1. Study Area

Poyang Lake, located in northern Jiangxi Province (115.817–116.767° E, 28.4–29.767° N), is a critical fluvial, throughput-type, and seasonal lake within the Yangtze River Basin. During the normal water period, the lake covers an area of approximately 3150 km^2^, expanding to over 4125 km^2^ in the wet season and shrinking to only 500 km^2^ during the dry season in winter. The Poyang Lake area lies within a hilly plain region surrounded by mountains, transitioning gradually from hills to plains that slope toward the lake, forming alluvial terraces and floodplains. Hills are distributed around the lake, while the surrounding areas are predominantly plains, largely composed of river valleys and lakeside lowlands. Poyang Lake experiences a typical subtropical monsoon climate, with southerly winds prevailing in summer bringing heat and abundant rainfall, and northerly winds dominating in winter resulting in lower temperatures and less precipitation. The average annual temperature is 17.6 °C, and the mean annual precipitation ranges between 1450 and 1550 mm, concentrated mainly from April to June [29]. The wetland vegetation in the lake area is characterized by rich plant diversity, complex geographical composition, and abundant widely distributed species, with Asteraceae, Poaceae, and Cyperaceae being the dominant families. During the wet season, the wetland exhibits typical lacustrine hydrological conditions. As the water level recedes, emerging mudflats at varying elevations give rise to well-developed wetland vegetation, extensive marshes, and mudflats, forming a mosaic landscape of rivers, lakes, and wetlands [30]. The site selected for Milu acclimatization is the Baishazhou Scenic Area (116.618400° E, 29.162275° N), an experimental zone within Poyang Lake National Wetland Park, located in Baishazhou Township, Poyang County, covering an area of approximately 0.3 km^2^.

### 2.2. Animals and Data Collection

**Figure 1:**
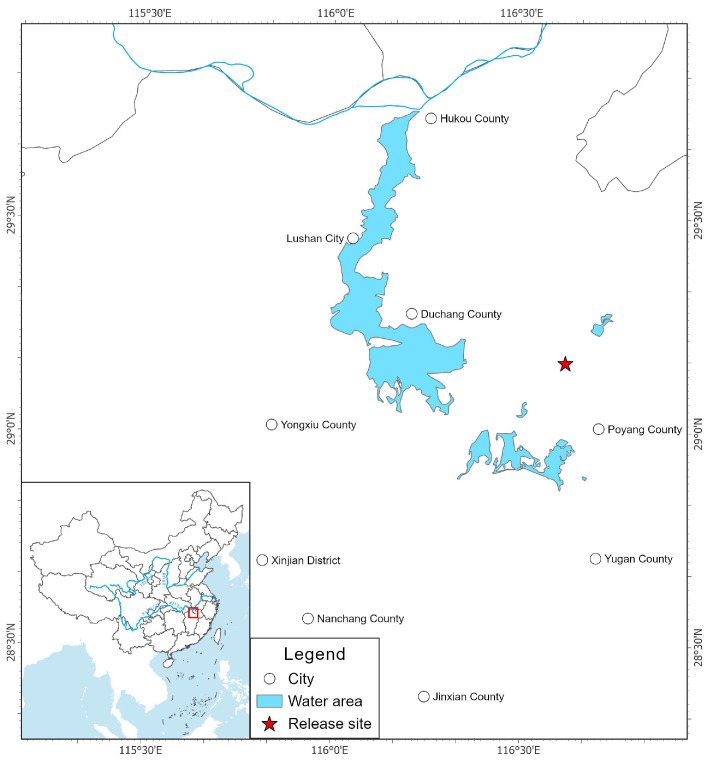
Study area of Poyang Lake.

The study subjects were Milu that were reintroduced to semi-natural enclosure of Poyang Lake in 2013 and 2018, and subsequently released into the wild in April 2018. In December 2013, 10 Milu were initially reintroduced into Poyang Lake Wetland Park for acclimatization from Beijing Milu Park where Milu were kept in captivity. In March 2018, an additional 30 individuals were translocated from Beijing Milu Park to the same site for short-term feeding, and one of the male individuals was fitted with GPS collar. On 3 April 2018, a total of 47 deer (24♂, 23 ♀) were released on-site into the wild of Poyang Lake area (Figure 1).

#### 2.2.1. Monitoring Milu Population and Acclimatization Process 

From December 2013 to March 2018, a dedicated breeder conducted daily artificial feeding management and monitored Milu behaviors during the acclimatization process. Records included health status, reproductive behavior, and other characteristics of the Milu population dynamics.

#### 2.2.2. Monitoring of the Released Milu Population Dynamics

From April 2018 to May 2025, the distribution and abundance of the released milu population within the Poyang Lake area (covering 9 counties and cities) were investigated using GPS collar tracking, field surveys, and feedback from local villagers. Location signals were transmitted every two hours from GPS satellite-tracking collar (HQAN40L, 800 g, 5-year battery life, solar-powered, Hunan Global Messenger Technology Co., Ltd., Changsha, China). Between 3 April 2018, and 31 May 2025, a total of 303 to 396 frontline rangers from wildlife protection stations in 9 counties and cities (including Poyang County, Yugan County, Nanchang County, Xinjian District, Yongxiu County, Duchang County, Jinxian County, Hukou County, Lushan City) around Poyang Lake, as well as from two nature reserves (Jiangxi Poyang Lake Nanji Wetland National Nature Reserve and Jiangxi Poyang Lake National Nature Reserve), conducted daily patrols and monitoring of the lake area. Survey tools included the use of monocular/binoculars, drones, and other equipment. Surveys were primarily conducted along lake embankments, with key areas further investigated via transects. Meanwhile, some information was gathered through visits to local villagers and reports received via phone calls from residents in the lake area when Milu entered into the farmland.

### 2.3. Data Analysis

Based on the water level dynamics of Poyang Lake, which were categorized into wet season (June to September) and dry season (October to April) [31], spatial analysis of collected data was conducted using ArcGIS 10.3 to map the interannual activity ranges of the Milu. Satellite image was obtained from https://earthexplorer.usgs.gov/ (accessed on 12 September 2025). Imagery for the dry and wet seasons was acquired on 28 December 2023 and 7 July 2024. To quantitatively reveal the spatial pattern and primary movement trend of the collared Milu, we conducted a directional analysis of their distribution using the Standard Deviational Ellipse tool in ArcGIS 10.3.

## 3. Results

### 3.1. Acclimatization Process of Milu in the Poyang Lake Basin

The establishment of the wild Milu population in Poyang Lake involved a process of initial acclimatization, secondary supplementary population, and eventual release into the wild. In May 2012, the Poyang Lake Milu Reintroduction Project was officially launched, initiating acclimatization site selection for Milu prior to release. After multiple field surveys, the Baishazhou Natural Wetland Exhibition Area within Poyang Lake National Wetland Park was ultimately chosen as a acclimatization site for its minimal human disturbance and abundant food resources such as aquatic plants. The acclimatization site was about 0.3 km^2^ with a closed iron fence.

On December 25, 2013, 10 Milu (3♂, 7♀) from the Beijing Milu Park were reintroduced to Poyang Lake. The acclimatization process was designed to prepare the captive-bred Milu for independent survival: (1) Habitat adaptation: the deer were allowed to freely explore the semi-natural enclosure to familiarize with the local climate, hydrology, and terrain of Poyang Lake. (2) Natural foraging induction: the dried alfalfa was added and concentrated feed was gradually reduced to facilitate their transition from provided feed to foraging on local plants. Simultaneously, during periods of winter food scarcity, various native plants such as reeds (*Phragmites australis*) and sedges (*Carex* spp.) were supplemented to stimulate their natural foraging behavior. (3) Social structure: in accordance with the herd-living nature of Milu, mixed-sex groups were maintained throughout the acclimatization period.

The first milu calf was born on 12 April 2014, followed by the birth of 3–4 calves annually in subsequent years. After nearly five years of acclimatization, the population reached 21 individuals by the end of 2017 (Table 1). These results demonstrated that the acclimatization was effective, the Milu gradually adapted to the Poyang Lake environment, showing significantly enhanced survival capacity. These results demonstrated that the systematic acclimatization was effective. The Milu gradually acclimated to the Poyang Lake environment, showing significantly enhanced survival capacity.

In March 2018, to accelerate the establishment of a wild population in the Poyang Lake basin and enhance genetic diversity by introducing new bloodlines, an additional 30 deer (17♂, 13♀) were reintroduced from Beijing Milu Park. In April 2018, 47 deer (24♂, 23♀) were selected and released on-site into the wild in the Poyang Lake basin.

### 3.2. Population Dynamics of Milu After Released in Poyang Lake

**Figure 2:**
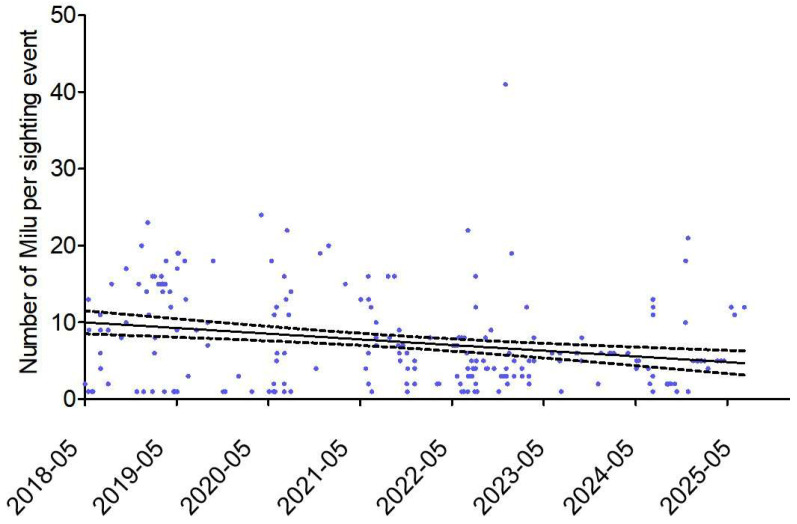
Number of Milu per sighting event in Poyang Lake from May 2018 to May 2025.

After the release, Milu deer were sighted a total of 238 times (including 10 occasions with an unknown number of individuals by local villagers), amounting to 1697 sighted individuals (Table 2). Among these, of the 952 individual records by sexual identification, there were 365 males and 587 females (a ratio of 1: 1.63). The mean number of individuals per sighting event was 7.28 ± 0.68. The observed population size showed a slight declining trend over the years (*R*^2^ = 0.063, *p* < 0.001) (Figure 2). From 2019 to 2023, the mean number of individuals per sighting event decreased from 11.26 to 5.13.

After the release of the Milu, a total of 52 newborn calves were observed, while 10 adult mortalities were recorded. Among these fatalities, 5 individuals (50%) died due to entanglement in abandoned fishing nets. The theoretical estimated population size of the Milu is 89 individuals. Additionally, 9 deer were rescued from abandoned fishing nets, and 12 deer were rescued after becoming trapped in irrigation channels by local residents, volunteers, and the police.

### 3.3. Distribution Characteristics of Milu After Released in Poyang Lake

**Figure 3:**
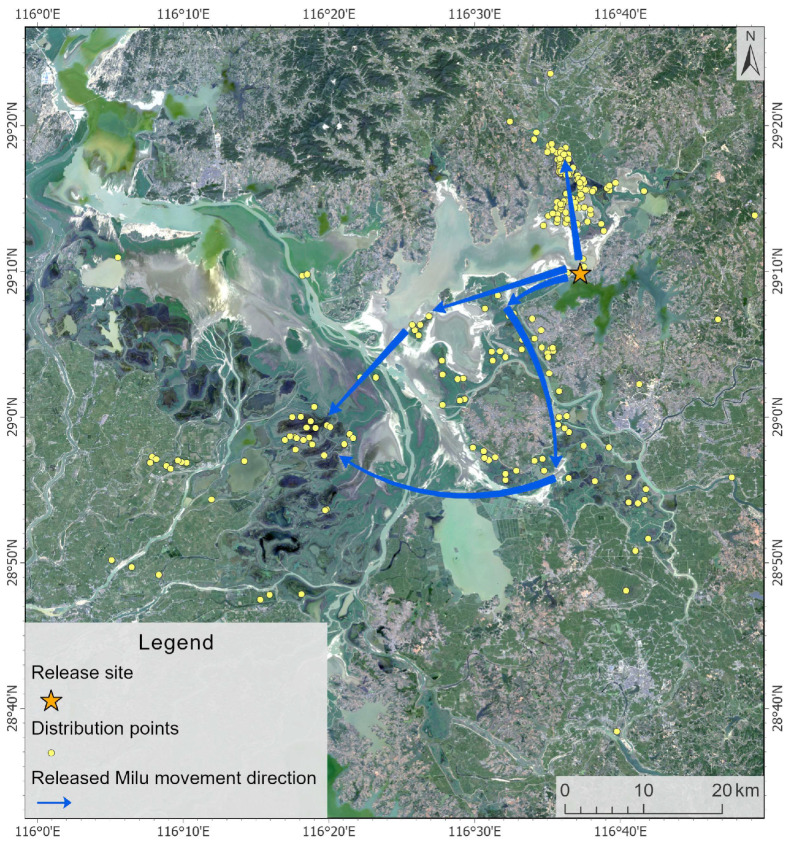
Post-release Dispersal Patterns of Milu in Poyang Lake.

**Figure 4:**
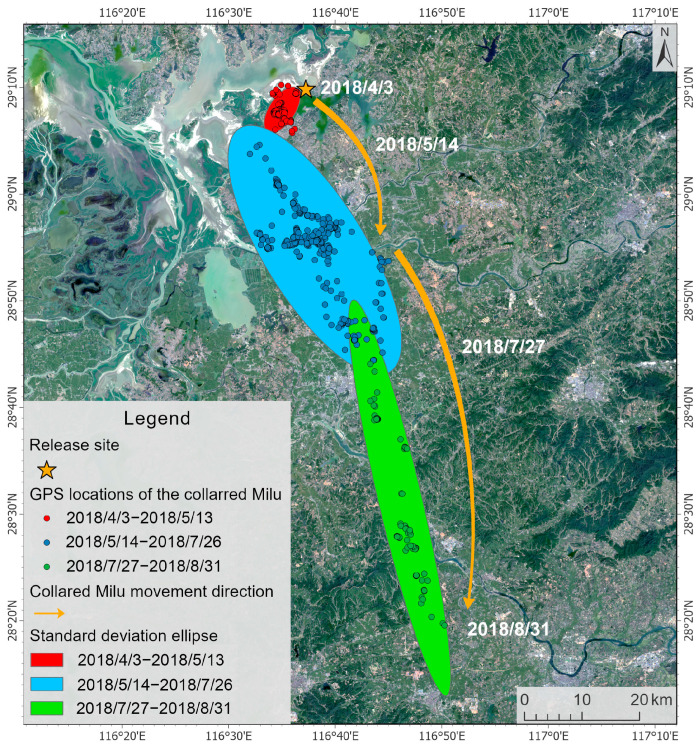
Distribution points and movement direction of the collared Milu.

The activity range of the Milu covered the entire eastern Poyang Lake, and only two individuals were observed in Yongxiu County, within the West Poyang Lake. The deer primarily inhabited areas in the northeast, including Yinbaohu Township, Shuanggang Town, and Lianhu Township in Poyang County, as well as the lake regions near Yufeng Township and Zhupao Mountain Island in Duchang County. They were also found in Nanji Wetland National Nature Reserve in Xinjian District of Nanchang City. After release, the Milu split into two groups, one population moved northeast along the eastern shore of Poyang Lake to Yinbaohu Township and Yaquehu Township in Poyang County, while the other migrated southward along the same shore to Shuanggang Township and Lianhu Township in Poyang County, eventually reaching Nanji Wetland National Nature Reserve (Figure 3). The collared deer moved to the southeast and finally arrived at Yingtan City, far from the lake area, 95.69 km away from the release site (Figure 4).

**Figure 5:**
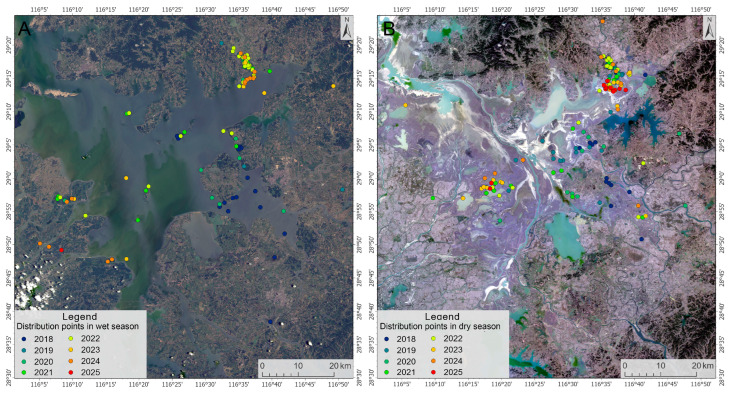
Distribution of Milu in Poyang Lake during wet season (**A**) and dry season (**B**) (April 2018–July 2025).

A total of 98 sighting events (41.18%) occurred during the wet season, and 140 sighting events (58.82%) were recorded in the dry season. After adapting to the local climate and environment, the deer migrated during the wet season to islands within the lake, as well as elevated areas such as surrounding hills and farmland—particularly in Yinbaohu Township of Poyang County and Jiangxiang Township of Nanchang County—where they occasionally caused damage to crops. Some individuals also exhibited upstream dispersal along rivers. During the dry season, the deer returned to shallow waters and grassland mudflats in the lake area (Figure 5).

## 4. Discussion

### 4.1. Establishment of the Milu Population in the Poyang Lake Basin and Factors Underlying Its Success

The reestablishment of a wild Milu population in Poyang Lake and the consistent recruitment of calves for eight consecutive years marked the success of the reintroduction project of this large mammal in Poyang Lake area. According to the Guidelines for Reintroductions and Other Conservation Translocations of IUCN [1], a systematic approach to reintroduction is essential: (1) a thorough feasibility assessment addressing habitat suitability, threats, and socio-economic factors; (2) careful selection of a source population; (3) a scientifically informed release strategy, which may employ either soft- or hard-release methods as appropriate; and (4) long-term post-release monitoring, coupled with adaptive management. Crucially, the guidelines emphasize that considerations of legal frameworks and stakeholder engagement must be integrated throughout the entire project cycle. The process of rebuilding the Milu population in Poyang Lake aligned with the IUCN’s practices for reintroducing endangered species. The Poyang Lake reintroduction project, spanning 14 years, was systematically divided into two key phases: acclimatization and field release. This model provides an important reference for the restoration of endangered species populations globally. First, Poyang Lake is a historical distribution area for Milu, and the acclimatization site was located in the experimental zone of a protected natural area with minimal human disturbance, effectively enhancing the survival ability of Milu in the wild. Second, the acclimatization site covered 0.3 km^2^, and the deer were managed using a semi-free-ranging approach, with supplemental feeding provided only during the winter when food was scarce.

The release into the wild involved 47 individuals, representing the largest foundational population ever used in a Milu reintroduction program, and was conducted in the expansive Poyang Lake area where food resources are relatively abundant—unlike a previous unsuccessful attempt in the Luanhe Upper River Nature Reserve in Hebei Province, which failed due to a small size number of release population (6 individuals), proximity to human settlements, and scarce food availability [26]. Releasing a large population helps mitigate extinction risks from uncertain factors, as initial release size is a key determinant of success in endangered species reintroductions, with larger groups generally exhibiting higher survival and establishment rates owing to three main mechanisms: greater genetic diversity reducing inbreeding depression [32], the Allee effect enhancing cooperation in foraging and defense [33], and improved demographic resilience against stochastic events such as disease or climate fluctuations [34]. For example, European bison (*Bison bonasus*) reintroductions showed that populations with over 50 individuals had significantly higher survival rates [35], and releases of Arabian oryx (*Oryx leucoryx*) in Oman demonstrated that larger groups (>100 individuals) achieved greater long-term stability [36].

Moreover, the successful establishment of a wild Milu population in Poyang Lake was inseparable from the Chinese government’s wildlife protection efforts, benefiting from a multidimensional synergy of legal frameworks, policies, media engagement, and public participation [37,38]. The improved Wildlife Conservation Law and Wetland Conservation Law provide solid legal safeguards for the deer and their habitats. The implementation of the Yangtze River Conservation Strategy has not only delineated key protected areas but also effectively reduced human disturbance through the ten-year fishing ban, while a complementary wildlife damage compensation insurance mechanism has alleviated human–wildlife conflicts. National and provincial mainstream media amplify ecological conservation awareness around key dates such as Wildlife Protection Publicity Month, International Biodiversity Day, World Wildlife Day, and World Wetlands Day through diverse campaigns, significantly raising public awareness of wildlife protection. Simultaneously, a grid-based protection system composed of professional rangers and volunteers covers towns and villages across the nine counties around the lake, conducting round-the-clock patrols that effectively curb poaching—as evidenced by the successful rescue of 21 stranded Milu.

### 4.2. The Distribution and Population of Reintroduced Milu in Poyang Lake

Our research had found that the migration routes of Milu vary seasonally in close correlation with food availability and water level fluctuations, demonstrating strong adaptability and survival strategies. After reintroduction, the deer dispersed northeastward along the eastern shore of Poyang Lake to the Yinbao Lake area and southward to the Nanji Wetland National Nature Reserve. After years of adaptation, their distribution and migration patterns now exhibited clear seasonal shifts driven by water level changes. During the flood season, the Milu migrated upstream along rivers, a behavior consistent with that of the Zhuzi River Milu population in East Dongting Lake [39]. This spatial distribution pattern aligned with habitat selection strategies observed in other reintroduced deer species. Notably, their movement to higher ground (e.g., hills and farmland) to avoid summer floods resembles the behavior of marsh deer (*Blastocerus dichotomus*) in Brazil’s Pantanal wetlands, which similarly retreat to forested highlands during seasonal flooding [40]. However, unlike North American wapiti (*Cervus canadensis*), which establish fixed winter ranges [41], the Poyang Lake deer display remarkable adaptability by returning to exposed grassland habitats in the lake area during the dry season—a trait shared by Eld’s deer (*Rucervus eldii*) adapted to floodplains [42].

The observed dispersal routes along the eastern shore underscore the critical role of landscape connectivity in reintroduction success. This aligned with findings from European bison reintroductions in Poland, where riparian corridors facilitated population expansion [43], but contrasts with the constrained dispersal of Milu in China’s Dafeng Reserve due to artificial waterways [26]. Their seasonal migrations between Poyang Lake and adjacent human-dominated landscapes exhibit ecological plasticity comparable to that of the rediscovered silver-backed chevrotain (*Tragulus versicolor*) in Vietnam [44]. However, their increasing use of farmland has led to growing human–wildlife conflicts—an issue particularly prominent in Japanese sika deer (*Cervus nippon*) populations [45]. Climate change-induced hydrological extremes may further compress suitable habitats, necessitating adaptive management strategies similar to those implemented for moose (*Alces alces*) in Minnesota wetlands [46].

Currently, the theoretical population size of Milu in Poyang Lake is estimated at 89 individuals. However, due to the extensive marshland terrain, field surveys are challenging to conduct thoroughly. Additionally, limited funding has restricted the use of long-endurance drone monitoring equipment. Thus, the actual population size of wild Milu in Poyang Lake requires further in-depth investigation.

### 4.3. Challenges in the Conservation of Milu in Poyang Lake

Despite the successful reintroduction of Milu deer to Poyang Lake, multiple challenges persist, including climate change, hydrological variability, and human disturbance. First, the summer flood season in Poyang Lake coincides with the peak rutting period (June and July) of Milu deer. Flooding forces the population to fragment into small groups, often resulting in spatial segregation of males and females. This disrupts optimal mating opportunities and consequently reduces pregnancy rates. GPS collar monitoring has documented cases where subadult males became isolated up to 50 km from the lake, entering mountainous areas—a phenomenon observed consistently over eight years. When flooding displaces deer 5–10 km into human-dominated landscapes, habitat barriers and anthropogenic pressures often prevent them from returning to the lake. Second, recent climate extremes in the Poyang Lake region—such as severe droughts in 2019, 2022, and 2025, and extreme flooding in 2020—have significantly impacted population dynamics. Calves and weakened individuals are particularly vulnerable to drowning during floods, as confirmed by field surveys. Prolonged flooding can also lead to food shortages and habitat loss, while extended droughts degrade wetland ecosystems, reducing the availability of key vegetation such as reeds and sedges. Similar challenges have been reported in the reintroduction of Yangtze finless porpoises, where altered hydrological conditions reduce fish resources and directly affect food availability [47,48]. Third, influenced by the East Asian monsoon, Poyang Lake exhibits pronounced seasonal flooding. Frequent flooding forces deer to migrate from the lake core to farmlands and human settlements along the shore, increasing human–wildlife conflict. Fourth, abandoned fishing nets frequently entangle Milu deer, causing injuries and fatalities. During the rutting season, males engage in distinctive vegetation-thrashing displays, which often lead to entanglement in discarded nets. Without human intervention, entangled deer typically die. Monitoring data indicate that entanglement accounts for 50% of documented deaths, and 9 of 21 rescued deer were males trapped in nets. Fifth, human disturbance remains frequent. During the dry season, exposed lakebeds attract increased fishing and tourist activities, disrupting deer behavior. Illegal off-road driving within the lake area continues despite bans, further compressing habitat and increasing stress on the population.

These challenges mirror those faced in other reintroduction programs globally. For example, species in the Yellowstone-to-Yukon region are forced into suboptimal habitats due to climate change [49]. European bison reintroduced in Poland experienced slow population growth and inbreeding due to human disturbance and habitat fragmentation [50]. Similarly, while the gray wolf reintroduction in Yellowstone National Park (1995–1997) is considered an ecological success, persistent human–wildlife conflicts—including the annual loss of 100–150 livestock animals (primarily cattle and sheep)—remain a key sustainability challenge [51]. The situation of the milu deer at Poyang Lake, however, differs in certain aspects. Notably, there are no natural predators for this population. Although spatial overlap with domestic cattle occurred during the flood seasons of the initial introduction period, the subsequent grazing ban in the lake area is attributed to the current rarity of interspecific competition.

## 5. Conclusions and Conservation Implications

This study systematically analyzes the outcomes and challenges of the Milu deer reintroduction project in Poyang Lake from 2012 to 2025. Through the initial acclimatization and the second phase of population reinforcements, a wild population has been successfully established, demonstrating the feasibility of reintroducing endangered species in lake ecosystems with frequent human activities. Key factors contributing to this success include large-scale releases (47 individuals), scientifically selected habitat (a 0.3 km^2^-acclimatization site), and a collaborative ‘government–media–public’ conservation mechanism. The deer exhibited distinct seasonal migration patterns, forming an ecological rhythm that moving out of the lake area during flood seasons and returning during dry seasons.

However, the population faces multiple threats, including extreme weather events, abandoned fishing nets, and human disturbances. Future efforts should enhance hydrological management, restrict illegal activities such as fishing and tourism within the lake area, and promote community-based conservation. Additionally, further reintroductions and wild releases should be conducted to consolidate and expand the wild Milu population in Poyang Lake, ultimately contributing to the establishment of a cross-regional protection network.

## Figures and Tables

**Table 1:** Population Status of Milu During Acclimatization Process.

Year	Population Size at the End of the Year	Number of Calves	Number of Deaths	Note
2013	9	-		The founder population consisted of 10 individuals, one of which (a male) died during transport due to stress syndrome.
2014	12	3	0	
2015	15	3	0	
2016	17	4	2	One male deer died from fighting, and one female deer died of digestive tract diseases.
2017	21	5	1	One female deer died of unknown causes.
2018	51	-	-	Secondary reintroduction (n = 30). First cohort (n = 47) released into the wild.

**Table 2:** Population Status of Milu Released in Poyang Lake.

Year	Number of Sighting Events	Number of Sighted Individuals	Mean Number of Individuals Per Sighting Event	Number of Calves	Number of Dead Deer	Number of Milu Rescued
2018	30	155	7.75	10	2	3
2019	39	439	11.26	12	1	2
2020	26	220	8.46	8	2	2
2021	29	208	7.17	5	3	1
2022	48	296	6.17	10	2	1
2023	23	118	5.13	2	0	0
2024	30	178	5.93	4	0	12
2025	13	83	6.38	1	0	0
Total	238	1697	7.28	52	10	21

## Data Availability

The data presented in this study are available on request from the corresponding authors.
